# Roles of microRNAs in Gastrointestinal Cancer Stem Cell Resistance and Therapeutic Development

**DOI:** 10.3390/ijms22041624

**Published:** 2021-02-05

**Authors:** Ga-Ram Hwang, John G. Yuen, Jingfang Ju

**Affiliations:** Department of Pathology, Renaissance School of Medicine, Stony Brook University, Stony Brook, New York, NY 11794, USA; ga-ram.hwang@stonybrook.edu (G.-R.H.); john.yuen@stonybrookmedicine.edu (J.G.Y.)

**Keywords:** cancer stem cells, microRNAs, cancer therapeutics, colorectal cancer, gastric cancer, pancreatic cancer, liver cancer

## Abstract

Resistance to cancer treatment is one of the major challenges currently faced when treating gastrointestinal (GI) cancers. A major contributing factor to this resistance is the presence of cancer stem cells (CSCs) in GI cancers (e.g., colorectal, pancreatic, gastric, liver cancer). Non-coding RNAs, such as microRNAs (miRNAs), have been found to regulate several key targets that are responsible for cancer stemness, and function as oncogenic miRNAs (oncomiRs) or tumor suppressor miRNAs. As a result, several miRNAs have been found to alter, or be altered by, the expression of CSC-defining markers and their related pathways. These miRNAs can be utilized to affect stemness in multiple ways, including directly targeting CSCs and enhancing the efficacy of cancer therapeutics. This review highlights current studies regarding the roles of miRNAs in GI CSCs, and efforts towards the development of cancer therapeutics.

## 1. Introduction

Cancer treatment regimens have advanced with the discovery of novel detection methods and therapeutics. As a result of these advances, cancer survival rates have continued to improve [1]. Unfortunately, despite these developments, the mortality rate in patients diagnosed with metastatic cancers has remained poor [1,2]. This can be seen by the continuous poor survival rates of patients with gastrointestinal (GI) cancers, such as colorectal cancer (CRC), pancreatic cancer, gastric cancer, and liver cancer [2]. One of the major treatment obstacles that has contributed to mortality is resistance to therapy [3,4].

Resistance to these therapies is generally defined as a poor response of the cancer to a given therapy [5]. Currently, there are a variety of therapies available for patients with metastatic cancer, including chemotherapy, radiotherapy, targeted therapy, and immunotherapy [1,3,6,7,8]. Depending on the patient’s condition, these therapies can also be used in combination to improve efficacy and patient survival [1]. However, despite novel discoveries in these therapies, cancer recurrence and resistance has still been observed [4]. Several mechanisms have been observed to contribute to a cancer’s resistance to therapy, including efflux of chemotherapeutics, resistance to apoptosis, and the repair response to DNA damage [5,6,9,10,11]. As tumor cells are highly heterogeneous, it has been suggested that cancer stem cells (CSCs) exhibit these characteristics and are largely responsible for cancer recurrence and resistance to therapy [3,6].

CSCs are cancer cells that have a “stem-like” nature of continuous self-renewal and tumor-initiating capacity [12,13,14]. It is important to note that although they are called cancer stem cells, this term only refers to how these populations exhibit characteristics similar to stem cells [12,15]. CSCs were first discovered in leukemia, and they were discovered to have acquired tumor-initiating capabilities, as seen by their ability to initiate tumor formation in mouse xenografts [13,14,16,17]. After the initial discovery of CSCs in leukemia, CSC populations were also discovered in solid tumor cancer types such as breast cancer [12,16,18]. In addition, CSC subpopulations, or cancer cells with stem-like qualities, have also been discovered in GI tumor types, including CRC, pancreatic cancer, gastric cancer, and liver cancer [2,16,19,20,21,22,23]. The stem-like nature of CSCs has been found to contribute to tumor growth, recurrence, and resistance to therapeutics [3,4,6,15,16,24]. Furthermore, the plasticity of the CSC state has been observed as a result of the surrounding CSC niche, thus adding an additional obstacle to therapeutic strategies [2,12,14]. As a result, there has been a growing amount of research that specifically identifies and targets CSCs, including the role of microRNAs (miRNAs) [25,26,27].

MiRNAs are a family of 21–25 nucleotide-long, double-stranded, non-coding RNAs (ncRNA). Similar to small interference RNAs (siRNAs), miRNAs are also involved in RNA interference (RNAi) through the degradation of messenger RNA (mRNA) and/or the inhibition of mRNA translation. Unlike siRNA’s specificity to one target per siRNA sequence via complementary binding, miRNAs bind to the 3′ untranslated region (UTR) of their target mRNA via complementary binding with the two- to seven-nucleotide-long seed region of the mature miRNA. As a result, this confers on miRNAs the ability to target multiple different targets per miRNA sequence [28,29,30]. Due to their role in regulation by RNAi, various cancer types, including CRC, pancreatic, gastric, and liver cancer, have been found to have dysregulated expressions of various miRNAs [26,28,30,31]. This includes the discovery of miRNAs that promote tumorigenesis, often referred to as oncomiRs, and miRNAs that inhibit tumorigenesis, often referred to as tumor suppressor miRNAs [30]. While many miRNAs have been demonstrated to inhibit tumor formation and/or growth, the tumor suppressor miRNAs mentioned in both the literature and in this review may not be a “classic” tumor suppressor gene that has been demonstrated to cause tumor formation after biallelic inactivation [32]. As a result of this dysregulation, some specific miRNA sequences have been proposed as novel biomarkers to identify CSCs [25,26]. Furthermore, miRNAs have also been found to affect the sensitivity of cancers and their respective CSCs to various therapeutics, including chemotherapy, radiotherapy, and targeted therapy [25,27]. In this review, we will highlight the role of miRNAs in GI CSCs and the impact of miRNAs in overcoming GI CSC resistance to therapeutics.

## 2. The Role of miRNAs in Colorectal Cancer Stem Cells

Among GI CSCs, CRC CSCs are one of the most well-studied types [33,34,35]. The intestinal epithelium is populated with stem cell niches, also known as intestinal crypts, that are responsible for the constant proliferation and differentiation of the epithelium. Due to this continuous proliferation and differentiation of cells, dysregulation in the proliferation and/or differentiation pathways within these cells can result in forming CRC CSCs [33]. Although the origin of CRC CSCs is still debated today, ultimately, among several other pathways, dysregulations within the Wnt [36], Notch, and TGF-β signaling pathways of the intestinal epithelial cells have all been found to contribute to the stemness of CRC CSCs [2,33,35,37]. As a result of these efforts, various genes associated with these pathways have been identified as potential biomarkers for CRC CSCs [2,34,38]. In addition to biomarkers directly associated with these pathways, general markers found in several different types of stem cells and CSCs, such as NANOG, OCT-3/4 (also known as POU5F1), and SOX2, are highly expressed in CRC CSCs [2,33,34,37,38]. In addition to these general markers for stemness, it has been well-documented that CD24, CD44, CD133, LGR5, ALDH1, CXCR4, DCLK1, and EpCAM (also known as ESA) can also be used to identify CRC CSCs [2,19,20,33,34,38,39,40,41]. Interestingly, CD44 has several known variants, with several variants—including CD44v6—contributing to the stemness of CRC CSCs [42].

It is important to note that several other biomarkers may be used to identify CRC CSCs, and the ones noted here only cover some of the more extensively studied markers.

Aside from the identification of CSCs, these markers are also found to be functionally active in CRC CSCs. For example, leucine-rich repeat-containing G protein-coupled receptor 5 (LGR5) expression correlates with the ability for long-term self-renewal and differentiation, known characteristics of CSCs, in human LGR5^+^ CRC cells [40,43]. Furthermore, the high expression of LGR5 has been found to contribute to CRC’s resistance to the chemotherapeutic drug 5-fluorouracil (5-FU) [44]. In addition to LGR5, EpCAM has also been identified as a marker for CRC CSCs [38]. The increased expression of EpCAM in CRC cells has been associated with CSC characteristics, such as tumor-initiating potential, colony formation, and long-term self-renewal [33].

The roles of miRNAs in CRC and their associated CSCs have also been extensively studied. Several miRNAs were identified to be upregulated in CRC CSC subpopulations, suggesting that they promote the stemness of CRC CSCs (Table 1) [45,46]. In both cell lines and patient-derived samples of CRC, miR-210 and miR-221 were generally found to be upregulated in CRC subpopulations that were enriched for CSC surface markers ALDH^+^ and CD44^+^ [47,48]. MiR-221-5p and miR-221-3p were also found to be upregulated in CRC cells that were sorted for CSC surface markers EpCAM^+^/CD44^+^. Furthermore, the overexpression of miR-221 in patient-derived xenografts (PDX) of CRC resulted in enhancing the formation of CRC 3D organoids. Conversely, downregulating miR-221 was found to inhibit proliferation, and reduce the formation of organoids and tumorigenic capacity. Downregulating the expression of miR-221 was found to also reduce the expression of CSC-associated markers LGR5, SOX2, and OCT-4 through the direct targeting of tumor suppressor QKI, a transcriptional target of p53 [49]. 

Likewise, miRNAs have also been identified as being downregulated in CRC CSCs, thus suggesting that they play a role in inhibiting the stemness of CRC CSCs [45,46]. Within the CRC ALDH^+^/CD44^+^ CSC subpopulation, the downregulation of miR-10b was observed. Interestingly, in the same study, although miR-18a was found to be upregulated in CRC cell lines compared to a non-stem and non-cancer colon cell line, miR-18a expression was downregulated after selection for CSC subpopulations, suggesting a possible role for maintaining stemness in CRC [47]. In CD44v6^+^ spheroid CRC subpopulations, miR-34a-3p, let-7f-1-3p, miR-101-3p and miR-200c-3p expressions were found to be downregulated. This CD44v6^+^ subpopulation was also found to have an upregulated expression of CSC markers OCT-4 and NANOG, along with the capacity for tumorigenesis in vivo [42].

In addition to observations in the downregulated expression of miRNAs in CRC CSC subpopulations, several miRNAs were also identified as inhibitors of CRC CSC stemness with direct targets that are involved in CSC stemness [45,46]. In CD133^+^/CD44^+^ CRC CSCs, miR-139-5p was found to inhibit activity within the Wnt signaling pathway through the transcription factor E2-2. By regulating the Wnt signaling pathway, miR-139-5p was found to regulate the stemness of CD133^+^/CD44^+^ CRC CSCs, including their self-renewal and tumorigenic capacity [55]. In a separate study, the expression of miR-140-5p was found to be downregulated in CD133^+^/CD44^+^ CRC CSCs. MiR-140-5p was found to target SMAD2 as part of the TGF-β/SMAD2/SMAD3 pathway and ATG12 in autophagosome formation, which suggests that miR-140-5p can regulate the stemness of CRC CSCs via the TGF-β pathway and through the autophagy of CRC CSCs. The downregulated expression of miR-140-5p was also observed in both primary and metastatic samples of patients with CRC. Furthermore, the transfection of CRC CSCs with miR-140-5p was found to inhibit the proliferation of spheroid cells and the invasion of CRC CSCs in vitro and in vivo [56]. Similarly, after transfection with miR-20b-5p, the subpopulation of CD133^+^/CD44^+^ CRC CSCs was found to be decreased in the CRC cell line HCT116. CSC-associated markers OCT-4, NANOG, SOX2, and NOTCH1 were also downregulated after transfection with miR-20b-5p in vitro. In addition, the tumorigenic capacity of HCT116 was found to be reduced after treatment with miR-20b-5p in vivo [51]. In spheroid CRC subpopulations that were positive for CD44, CD133, CD166, and CD24, the expression of miR-133b was found to be significantly downregulated compared to their respective parental cell lines. MiR-133b was found to reduce stemness by directly downregulating the expression of a methyltransferase, disruptor of telomeric silencing 1-like (DOT1L), which was observed to induce expression of OCT-3/4, NANOG, and SOX2. In turn, transfecting these spheroid subpopulations with miR-133b was found to reduce the ability to form spheroid colonies and stemness within these subpopulations, by downregulating the expression of CSC-associated surface markers CD44, CD133, CD166, and CD24. Furthermore, the expressions of CSC-associated markers OCT-3/4, NANOG, and SOX2 were also found to be downregulated after transfection with miR-133b [54]. In CRC subpopulations resistant to the chemotherapeutic Cetuximab, the relative expression of miR-302a was found to be significantly lower compared to non-resistant populations, both in in vitro cell lines and in an in vivo PDX model. Furthermore, in vitro transfection with miR-302a was found to inhibit the expression of the CSC marker CD44 by directly targeting the 3′UTR sequence of CD44. In addition to CD44, transfection with miR-302a was also found to downregulate the expression of other CSC markers, including SOX2, NANOG, EpCAM, CD133, and CD166. The ability to form spheroid colonies was also inhibited by miR-302a. In patient tissue samples of primary and metastatic CRC, the expression of miR-302a was found to be inversely correlated with the expression of CD44 [58]. Through the screening of putative targets for the CRC CSC-associated marker KLF5, miR-4711-5p was identified. Including KLF5, miR-4711-5p was found to downregulate the expression of CSC-associated surface markers CD44v9 and additional CSC-associated markers LGR5 and BMI1 in vitro, thus suggesting that miR-4711-5p suppresses stemness. In addition, miR-4711-5p was found to inhibit the formation of spheroid cells [59].

## 3. The Role of miRNAs in Pancreatic Cancer Stem Cells

Pancreatic cancer is a particularly deadly form of GI cancer with a 5-year survival rate of about 9% [60]. Pancreatic ductal adenocarcinoma (PDAC) is the most prevalent form of pancreatic cancer, making up about 90% of all pancreatic cancer cases. PDAC is largely defined by its desmoplastic stromal environment that can act both as an extrinsic cause of drug resistance and as a direct mediator of cancer progression. This tumor–stroma crosstalk is an active area of research, and its precise mechanisms are only beginning to be elucidated. 

A key population of these stromal cells includes pancreatic stellate cells (PSC) that contribute to the development of precancerous pancreatic intraepithelial neoplasias (PanINs), and the later facilitation of pancreatic cancer aggression via autocrine and paracrine signaling [61]. PSCs secrete embryonic morphogens Nodal/Activin, and were found to promote the sphere-forming ability of CSCs, suggesting that PSCs play a role in establishing a CSC niche in pancreatic cancer [62,63]. TGF-β is a cytokine that has also been implicated in both the early pathogenesis of pancreatic cancer and its later progression and metastasis. In normal pancreatic cells, TGF-β has a tumor suppressor function, inhibiting cell cycle progression [62]. In advanced disease, however, the dysregulation of the TGF-β pathway activates tumorigenic pathways such as PI3K/Akt and Ras/Erk. Over 50% of PDAC tumors have mutations of SMAD4, a key mediator of TGF-β signaling [64]. The TGF-β pathway is increasingly associated with stemness in pancreatic cancer, promoting the number of CD133^+^ pancreatic CSCs and upregulating stemness-related genes such as CD24, NANOG, and SOX2 [65,66,67]. Similarly, DCLK1 is not only important in early tumorigenesis and in tumor progression, but also in normal pancreatic injury-induced regeneration [68]. Elevated levels of DCLK1 and acetylated α-tubulin (AcTub) are found in human and mouse models of PanINs. These DCLK^HI^AcTUB^HI^ cells were shown to have increased tumor-initiating abilities both in an in vitro tumor sphere model and in an in vivo tumor formation model [69]. 

Since the identification of self-renewing, tumor-initiating pancreatic cancer cells in 2007, the identification of universal pancreatic CSC markers has not been clear-cut. Common CSC markers such as CD24, CD44, and CD133 have been repeatedly reported to be found on pancreatic CSCs [21,70]. Additional markers such as 

ALDH, c-Met, CXCR4, and EpCAM have also been associated with pancreatic CSC populations [70,71,72].

Pancreatic CSCs have also been shown to be regulated by miRNAs (Table 2) [73]. A common tumor suppressor miRNA, miR-34, directly targets the pancreatic CSC marker c-Met. CD44^+^/CD133^+^ pancreatic CSCs show a loss of miR-34 expression and an increased expression of genes involved in CSC maintenance, namely NOTCH1/2 and Bcl-2, direct targets of miR-34. Restoring miR-34 expression reduces the expression of CD44 and CD133 in vitro, and inhibits tumor formation in vivo. Lastly, the restoration of miR-34 sensitizes pancreatic cancer cells to docetaxel and gemcitabine [74].

As in other CSC populations, EMT activator ZEB1 dysregulates the expression of the miR-200 family, which normally suppresses stem cell factors such as BMI-1. These key markers for stemness, such as BMI-1 expression, are correlated with poorer prognosis in pancreatic cancer patients that have undergone surgical resection [82]. The miR-15 family regulates key stemness markers such as BMI-1 and DCLK1 in pancreatic cancer. The overexpression of miR-15a in pancreatic cancer cells reduces the expression of BMI-1, and similarly, the overexpression of miR-195 (a member of the miR-15 family) inhibits DCLK1 expression [75,77]. Dysregulation of the miR-200 family is also associated with stemness in pancreatic cancer. Notably, the overexpression of miR-200a was also found to decrease the expression of CD24, CD44, and EpCAM, and downregulate the EMT markers N-cadherin, ZEB1, and vimentin [78]. Furthermore, the knockdown of DCLK1 results in the upregulated expression of miR-200a [79]. MiR-205 is downregulated in pancreatic cancer, and in an in vitro model, the overexpression of miR-205 decreased the expression of both general stemness markers such as OCT-3/4 and NANOG, and more specific pancreatic CSC markers such as CD44 and ALDH1 [80]. 

In contrast to tumor suppressor miRNAs, miR-1246 has oncogenic properties and is correlated with a poor prognosis in patients with pancreatic cancer. In vitro, gemcitabine-resistant cell lines express higher levels of miR-1246 and have increased CD44^+^/CD24^+^ cell populations. The persistent expression of miR-1246 increases the tumor’s sphere-forming capability, and the gene set enrichment analysis of these cells showed enrichment of stemness pathways. In an in vivo mouse model, miR-1246 expression conferred resistance to gemcitabine [81]. Similarly, the expression of miR-135b was found to be upregulated in CD44^+^/CD24^+^/EpCAM^+^ CSCs. Inhibiting miR-135b with an antisense oligonucleotide, anti-miR-135b, decreased the expression of stemness markers such as NANOG, ALDH1, SOX2, and OCT-4 [76].

## 4. The Role of miRNAs in Gastric Cancer Stem Cells

Tumorigenesis of gastric cancer originates from the epithelial cells of the stomach. Similar to CRC, CSCs have also been identified within gastric cancer cell populations [22,83]. As a result, the dysregulation of the Wnt and TGF-β signaling pathways has been observed as well [37]. Similar to CRC CSCs, surface markers such as CD24, CD44, CD133, EpCAM, and LGR5 have been used to identify gastric CSCs [2,37,38,84]. CD44^+^ gastric cancer cell populations have been found to exhibit characteristics of CSCs, such as self-renewal, spheroid colony formation, and tumorgenicity in vivo [85]. Intracellular markers such as ALDH, NANOG, OCT-3/4, SOX2, and SOX9 are highly expressed in gastric CSCs as well. Although it is still controversial, it has been suggested that the increased expression of SOX2 results in a poorer prognosis in gastric cancer [2,22,37,38].

In addition to markers that are common with CRC CSCs, surface markers such as CD90 and CXCR4 have been used to identify gastric CSCs [38,84]. Similar to the high expression of CD44, the increased expression of CD90 has been observed in spheroid populations of gastric cancer cells along with a tumorigenic capacity in vivo [84,86]. Furthermore, within induced spheroid populations of gastric cancer cells, there is a positive correlation between the increased expression of CXCR4 and the ability for self-renewal and differentiation [84,85].

Several miRNAs have been identified in gastric CSCs, including those with targets that are associated with the TGF-β signaling pathway (Table 3). After sorting for the CSC-associated surface marker CD44 in vitro, the expressions of miR-106b and miR-196a-5p were observed to be upregulated in comparison to CD44^-^ gastric cancer cells [87,88]. miR-106b was found to directly target and downregulate the expression of the TGF-β pathway inhibitor SMAD7, thus activating the TGF-β signaling pathway [87]. Meanwhile, miR-196a-5p was found to target the co-SMAD, SMAD4 [88]. The loss of SMAD4 has been implicated in tumor. 

Progresssion to a more invasive phenotype, and poor differentiation, which has also been observed in patients with gastric cancer [96,97]. After the induction of the TGF-β signaling pathway via TGF-β1 in gastric cancer in vitro, the downregulation of miR-200a has also been observed. With the downregulation of miR-200a, gastric cancer cells were observed to have a correlation with the upregulation of EMT-associated transcription factors ZEB1 and Snail, thus acquiring the CSC characteristics of invasion and migration [91].

Aside from the TGF-β signaling pathway, the dysregulated expression of miRNAs has also been found to be associated with the Wnt signaling pathway, thus promoting the stemness of gastric CSCs. From spheroid gastric cancer cells, miR-216a-3p has also been identified to be downregulated by BRD4. BRD4 is overexpressed within spheroid gastric cancer cells, and downregulates the expression of miR-216a-3p by methylating its promoter region. Furthermore, transfection with miR-216a-3p has been observed to downregulate the expression of its direct target Wnt3a of the CSC-associated Wnt signaling pathway [93].

In addition to these signaling pathways, miRNAs associated with other pathways, such as cell cycle progression, have also been identified. For example, miR-26a has been observed to be downregulated in gastric cancer and has been identified as regulating stemness in gastric CSCs by downregulating HOXC9. HOXC9 has been suggested to promote the CSC phenotype in gastric cancer cells in vitro, as observed by the downregulation of CSC-associated surface markers CD44 and EpCAM, and CSC-associated markers SOX2 and OCT-4, after the knockdown of HOXC9. In addition, the overexpression of miR-26a reduces the self-renewal capacity of spheroid cells and the invasive capacity of GC CSCs [89].

## 5. The Role of miRNAs in Liver Cancer Stem Cells

Liver cancer consists of several subtypes, with hepatocellular carcinoma (HCC) being the most common subtype, which will be the main focus of this review [98,99]. Similar to CRC and gastric cancer, dysregulated expression in pathways such as the Wnt, Notch, and TGF-β pathways has been found to contribute to the stemness of HCC CSCs [99,100,101]. As a result, surface markers that have been used to identify other CSCs—such as CD24, CD44, CD90, CD133, and EpCAM—have been used to identify HCC CSCs, and intracellular markers, such as NANOG, OCT-3/4, and SOX2, have been used to identify HCC CSCs as well [23,38,98,99,101,102].

Aside from common CSC markers, specific HCC CSC markers, including OV-6 and cell surface calcium channel α2δ1, have been identified [98,99,100,101,103]. OV-6 is a marker that has been used to identify hepatic stem cells. In addition to hepatic stem cells, the expression of OV-6 has been observed in HCC, and a high expression of OV-6 has been found to correlate with CSC traits such as long-term self-renewal, differentiation and tumorigenic capacity [100,101,104]. The cell surface calcium channel α2δ1 has also been used to identify HCC CSCs. HCC cells expressing α2δ1 have been found to have tumorigenic potential, and HCC cells expressing α2δ1 have been found to also express other HCC CSC markers such as CD133 and EpCAM [98,99,103]. It has also been suggested that alpha-fetoprotein (AFP) may be used as a marker for HCC CSCs, as seen by elevated levels of stem cell markers and EpCAM in AFP^+^ patient-derived tumors [105].

Similar to the previously mentioned types of GI CSCs, the dysregulated expression of miRNAs has been identified after selecting for HCC CSC subpopulations (Table 4) [106]. MiR-30e-3p is negatively correlated with EpCAM expression in patients with HCC. In addition, miR-30e-3p was observed to downregulate the expression of the CSC-associated surface markers AFP and EpCAM in HCC in vitro. Furthermore, it was observed that overexpressing miR-30e-3p decreased spheroid colony formation in HCC cells, and conversely, silencing miR-30e-3p expression increased the number of HCC spheroid cells [107]. After sorting HCC cells with either the CSC-associated surface marker CD133 or EpCAM. 

miR-194 was found to be downregulated in both subpopulations. Conversely, stably overexpressing miR-194 in HCC resulted in the reduced expression of CSC-associated markers CD133, CD24, EpCAM, and CD90, and it reduced the number of HCC spheroid cells. In addition, miR-194 was found to directly target and downregulate the expression of RAC1, which was previously identified as being involved in EMT. Patients with low expression levels of miR-194 in HCC were found to have poorer prognosis [109]. In HCC CD133^+^/CD13^+^ spheroid CSCs, miR-1305 was found to inhibit spheroid formation and self-renewal in vitro and inhibit tumorigenic capacity in vivo. miR-1305 was able to reduce the stemness of HCC CSCs by downregulating its direct target UBE2T, which is upregulated in HCC CSCs and promotes stemness via the Akt-signaling pathway [113]. In HCC CD24^+^/OV6^+^ spheroid CSCs, miR-613 was downregulated when compared to its adherent counterparts. The downregulated expression of miR-613 was also observed in patients with recurrent HCC. Conversely, the overexpression of miR-613 in stably transfected HCC cells was found to reduce the formation of spheroid cells along with a reduced expression of several CSC-associated markers, including NANOG, OCT-4, and SOX2. MiR-613 was found to downregulate the stemness of HCC CSCs by downregulating its direct target SOX9, a CSC-associated marker [112].

In addition to downregulated miRNAs, several miRNAs have been identified to be upregulated in HCC CSCs as well [106]. The upregulation of miR-589-5p was observed in HCC compared to normal liver cells in vitro, and the upregulation of miR-589-5p was found to correlate with a poorer prognosis in patients with HCC. Several targets associated with the JAK/STAT3 signaling pathway were identified to be direct targets of miR-589-5p, including SOCS2, SOCS5, PTPN1, and PTPN11. As a result, the upregulation of miR-589-5p was found to promote HCC stemness by upregulating the expression of CSC-associated surface marker CD133 and CSC-associated markers NANOG, BMI-1, OCT-4, and SOX2, and promoting tumorigenesis in vivo [111]. MiR-181 is upregulated in the liver during embryonic development, and is thought to affect stemness due to its effects on the Wnt signaling pathway, namely on its targets GATA6, CDX2, and NLK. Altered miR-181 expression has been proposed as a predictive marker for HCC. In vitro, miR-181 was found to be elevated in EpCAM^+^ and CD44^+^/CD24^+^/CD90^+^ HCC stem cells [108].

## 6. The Role of miRNAs in Developing Therapeutics Targeting GI CSCs

In addition to the critical role miRNAs play in regulating the stemness of GI CSCs, via a number of unique mechanisms described above, some of these miRNAs have also been identified as having a direct role in either promoting resistance or enhancing sensitivity to several avenues of cancer therapeutics, including chemotherapy, radiotherapy, and immunotherapy [25,27,114,115]. As a result, modulating miRNAs expression, either through silencing or overexpressing specific miRNAs, in cancer therapeutics is a promising avenue to explore.

Several miRNAs have been observed to be upregulated in GI CSCs, suggesting that these miRNAs promote stemness. Resistance to chemotherapeutics is also a characteristic of CSCs, and likewise, a positive correlation with stemness-promoting miRNAs has been observed. For example, in gastric cancer, the upregulation of miR-193-3p has been found to increase the resistance of gastric cancer to 5-FU and cisplatin, which are commonly used to treat patients with gastric cancer [90]. As previously mentioned, the upregulated expression of miR-1246 confers resistance to gemcitabine in an in vivo model of pancreatic cancer [81]. This response also suggests that silencing these stemness-promoting miRNAs can improve sensitivity to therapeutics. In the previously mentioned miR-193-3p, inhibiting the expression of miR-193-3p in gastric CSCs has been found to sensitize CD44^+^ gastric CSCs to cisplatin [90]. In HCC CSCs, treatment with anti-miR-589-5p enhances the sensitivity of HCC CSCs to doxorubicin both in vitro and in vivo. Furthermore, anti-miR-589-5p was found to downregulate the expression of anti-apoptotic proteins Bcl-2 and Bcl-xL [111]. In addition to enhancing sensitivity to therapeutics, silencing stemness-promoting miRNAs could also be used as a therapeutic to inhibit proliferation. In CRC CSCs, transfection with anti-miR-221 was found to reduce proliferation and increase apoptosis in vitro. Transfection with anti-miR-221 was also found to reduce tumor growth in a PDX model of CRC in vivo [49]. Meanwhile, in pancreatic cancer, treatment with anti-miR-135b suppressed tumor growth in vivo [76]. Similarly, treatment with anti-miR-181 decreased the population of EpCAM^+^ HCC stem cells in vivo [108]. Silencing stemness-promoting miRNAs through anti-miRs can therefore be used to enhance sensitivity to chemotherapeutics, and can be explored as an approach to cancer therapeutics.

Aside from stemness-promoting miRNAs, several miRNAs have also been identified to be downregulated in GI cancers and in their respective CSCs. For example, in CRC CSCs resistant to chemotherapeutics 5-FU and oxaliplatin, several miRNAs were found to be downregulated, including miR-34a-3p, let-7f-1-3p, miR-101-3p and miR-200a-3p [42]. Similar to the therapeutic potential of silencing stemness-promoting miRNAs, restoring the expression of these downregulated miRNAs has been demonstrated to be a promising cancer therapeutic, and can enhance the sensitivity of GI cancers and their respective CSCs to other cancer therapeutics. In CRC, treatment with a miR-129-5p mimic has been found to promote apoptosis and enhance sensitivity to 5-FU [52]. In CRC CSCs, treatment with a miR-133b mimic has been found to enhance sensitivity to 5-FU and oxaliplatin [54]. In addition, a miR-148a mimic enhanced the sensitivity of CRC CSCs to cisplatin and promoted apoptosis [57]. As previously mentioned, treatment with miR-34 sensitizes pancreatic cancer cells to chemotherapeutics docetaxel and gemcitabine [74]. In an in vivo model of pancreatic cancer, the overexpression of miR-205 in mouse xenografts sensitizes the tumors to gemcitabine treatment [80]. Meanwhile, treatment with miRNA mimics miR-876-3p and miR-524-5p was found to enhance the sensitivity of gastric CSCs to cisplatin [94,95]. In chemotherapeutic sorafenib-resistant HCC cells, the overexpression of miR-194 and miR-613 was found to re-sensitize these HCC cells to sorafenib and promote apoptosis [109,112]. The overexpression of miR-613 was also found to re-sensitize cisplatin-resistant HCC cells to cisplatin as well [112]. 

Although restoring the expression of downregulated miRNAs is promising as a cancer therapeutic, one of the major obstacles that is currently encountered is toxicity associated with the delivery of these miRNAs [25,26,115]. A liposomal miR-34 mimic, MRX34, was developed for the treatment of solid cancers, and underwent Phase I clinical trials. The trial was unfortunately terminated in the United States due to immune-related serious adverse events, potentially associated with the miR-34 mimic delivery vehicle, but nonetheless represents a major effort in both miRNA-based therapeutics and in CSC therapeutics [25,114]. 

Several other miRNA-based cancer therapeutics have reached Phase 1 or Phase 2 clinical trials; however, none have entered Phase 3 [116,117,118]. The delivery of RNA-based therapies remains a major therapeutic hurdle [25,26,114,115]. As a result, several strategies have been developed to address this issue. One major strategy that has been developed to address this issue is the development of nanoparticle delivery vehicles, including lipid-based nanoparticles and gold nanoparticles [115]. Using micelle-based nanoparticles, a miR-200c mimic was successfully delivered to a gastric cancer cell line. As a result, it was found that miR-200c could enhance radiotherapy in gastric cancer [92]. Likewise, gold nanoparticles loaded with anti-miR-221 were used to deliver the anti-miR to HCC cells in vitro. Using these nanoparticles, it was also found that anti-miR-221 could enhance the sensitivity of HCC cells to sorafenib [110]. Another notable strategy to address the issue with miRNA delivery has been the modification of miRNA itself [26,30,119]. Some modifications include modifying the miRNA sequence with a 2′-*O*-Me group or with a 2′-F group to improve stability [26,30,119]. 

**Figure 1:**
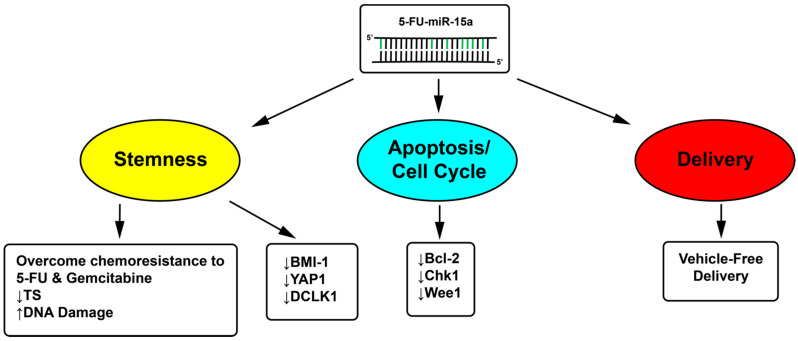
Modification of tumor suppressor miRNA with 5-FU as a novel miRNA-based therapeutic strategy. The guide strand of tumor suppressor miR-15a can be modified by replacing the uracils with a chemotherapeutic nucleoside analog, 5-FU (green), to make 5-FU-miR-15a. The passenger strand remains intact to minimize off-target effects. The 5-FU-miR-15a has several unique properties derived by integrating the therapeutic powers of miR-15a with 5-FU to impact multiple targets/pathways, such as stemness, apoptosis and cell cycle in CRC and PDAC (down arrows represent downregulated expression and up arrow represents increased effect). In addition, 5-FU-miR-15a can be delivered without the use of a delivery vehicle. This novel modification strategy can potentially be applied to other tumor suppressor miRNA candidates and cancer types.

In order to overcome the challenge of delivery with miRNA therapeutics, our recent studies took a notable approach by modifying the guide strand of tumor suppressor miRNA sequences with 5-FU. 5-FU is a nucleoside analogue for uracil, and therefore, it is possible to modify miRNAs by replacing the bases containing uracil on the mature miRNA sequence with 5-FU. 5-FU-based chemotherapy is still a main component of the therapeutic regimens against GI cancers, which operates by inhibiting the target enzyme, thymidylate synthase (TS), to cause DNA damage. This approach integrates the therapeutic powers of the tumor suppressor functions of both the miRNA and 5-FU into one entity (Figure 1). 5-FU-modified miRNA mimetics are a potent therapeutic candidate to inhibit multiple oncogenic targets and pathways via a number of unique mechanisms, such as stemness, apoptosis, cell cycle, and other key DNA-damaging enhancement mechanisms. As a result, this modification provides the miRNA with enhanced stability and potency, and notably, permits delivery without a delivery vehicle, as seen with 5-FU-miR-129 and 5-FU-miR-15a [50,53,75]. Treatment with 5-FU-miR-129 has been found to inhibit proliferation in CRC in vitro, and both proliferation and metastasis in vivo. Furthermore, 5-FU-miR-129 is effective at inhibiting the formation of spheroid CRC populations and eliminating 5-FU-resistant CRC CSCs. It is important to note that target specificity for 5-FU-miR-129 is retained, as seen by the downregulation of miR-129 targets Bcl-2 and thymidylate synthase (TS) [53]. Similar to 5-FU-miR-129, 5-FU-miR-15a has also been found to inhibit proliferation in CRC and pancreatic cancer in vitro, and both proliferation and metastasis in vivo. In addition, 5-FU-miR-15a was also found to retain target specificity by the downregulation of miR-15a target Yap1, and CSC-associated markers BMI-1 and DCLK1 [50,75]. Interestingly, in pancreatic cancer, 5-FU-miR-15a alone and in combination with gemcitabine has shown inhibition of metastatic tumor growth in an in vivo metastasis model, suggesting that modified miRNAs could also be an effective therapeutic used in conjunction with additional chemotherapeutics [75]. In addition, both 5-FU-miR-129 and 5-FU-miR-15a have IC^50^ values roughly 200-fold lower than the IC^50^ of 5-FU [50,53,75].

## 7. Conclusions

In this review, we have highlighted recent work in identifying GI CSCs through the dysregulated expressions of miRNAs and the roles that they play in the treatment of these CSCs. It is important to note that the roles of miRNAs and CSCs mentioned in this review are not limited to GI CSCs. MiRNAs have also been identified to play a role in the CSCs of other cancer types, including lung cancer and breast cancer [120,121,122]. This also includes miRNA-based therapeutics, with several miRNA-based therapeutics currently in Phase I/II clinical trials. In addition, success has also been observed with 5-FU-modified miRNAs as a cancer therapeutic outside of GI cancers, as seen by 5-FU-modified miR-489 in triple-negative breast cancer [123]. This suggests a therapeutic potential for 5-FU-modified miRNAs as a potential platform drug development technology for both GI cancers and other cancer types.

With regard to GI CSCs, an important caveat to note is the plasticity of CSCs and their surrounding niche. Unlike hematopoietic CSCs and CSCs in glioblastoma, CSCs from solid tumors, including GI cancers, have been found to exhibit plasticity in the CSC state between the CSCs and their surrounding niche [14]. As a result, it has been suggested that cancer therapeutics targeting CSCs need to address this issue as well. Fortunately, due to the pleiotropic nature of miRNAs, it is possible that miRNAs can address the stemness of CSCs and the plasticity of their surrounding niche. However, future studies will need to be done exploring this hypothesis.

While we focused on nanoparticles and RNA modifications as methods for miRNA delivery, there are several other promising miRNA delivery technologies, including miRNA-packaged extracellular vesicles and bacterial minicells [116,124,125,126,127]. There have also been major advancements in the broader field of RNA-based medicine with the recent success of the mRNA-based vaccines against SARS-CoV-2. Both vaccines from Pfizer and Moderna utilize a lipid-nanoparticle delivery mechanism, and have demonstrated both the safety and the efficacy of an RNA-based drug [128,129].

Cancer stem cells in GI cancers have proven to be a major obstacle in cancer therapeutics due to their role in recurrence and resistance to cancer therapeutics. Therefore, it is important to develop a therapeutic strategy that can adequately address this obstacle. Fortunately, studies have shown that miRNAs play a role in both identifying and treating GI CSCs due to their dysregulated expression. This demonstrates the potential of miRNA-based therapeutics targeting CSCs, and warrants future studies.

## Figures and Tables

**Table 1:** Summary of microRNAs (miRNAs) regulating colorectal cancer stem cell (CSC) growth and/or stemness.

miRNA	Expression in CSCs	Relation to Stemness	Reference
let-7f-1-3p	Downregulated	Downregulated in CD44v6^+^ cells	[42]
		Downregulated in 5-fluorouracil (5-FU) and oxaliplatin-resistant CRC^1^	
miR-10b	Downregulated	Downregulated in ALDH^+^/CD44^+^ cells	[47]
miR-15a	Downregulated	Targets BCL2, BMI1, YAP1 and DCLK1	[50]
		5-FU-modified mimic promotes apoptosis, inhibits invasion and overcomes resistance in CSC ^1^	
miR-18a	Downregulated	Upregulated in CRC, but downregulated in CRC CSCs	[47]
miR-20b-5p	Downregulated	Inhibits proliferation of spheroid cells, invasion and tumorigenic capacity	[51]
miR-34a-3p	Downregulated	Downregulated in CD44v6^+^ cells	[42]
		Downregulated in 5-FU and oxaliplatin-resistant CRC	
miR-101-3p	Downregulated	Downregulated in CD44v6^+^ cells	[42]
		Downregulated in 5-FU and oxaliplatin-resistant CRC	
miR-129	Downregulated	Promotes apoptosis and inhibits proliferation	[52,53]
		Targets BCL2 and E2F3	
		Enhances sensitivity to 5-FU ^1^	
		5-FU-modified mimic promotes apoptosis, inhibits invasion and overcomes resistance in CSC ^1^	
miR-133b	Downregulated	Downregulated in CD44^+^/CD133^+^/CD24^+^/CD166^+^ cells	[54]
		Targets DOT1L which promotes expression of OCT-3/4, NANOG, and SOX2	
		Enhances sensitivity to 5-FU and oxaliplatin ^1^	
miR-139-5p	N/A	Overexpression leads to loss of stemness in CD133^+^/CD44^+^ CSCs	[55]
		Targets E2-2 which promotes Wnt signaling pathway	
miR-140-5p	Downregulated	Inhibits proliferation of spheroid cells and invasion	[56]
		Targets Smad2 which promotes TGF-β pathway	
		Targets ATG12 which promotes autophagy	
miR-148a	Downregulated	Promotes apoptosis	[57]
		Enhances sensitivity to cisplatin ^1^	
miR-200c-3p	Downregulated	Downregulated in CD44v6^+^ cells	[42]
		Downregulated in 5-FU and oxaliplatin-resistant CRC	
miR-210	Upregulated	Upregulated in ALDH^+^ and CD44^+^ cells	[47,48]
miR-221	Upregulated	Upregulated in ALDH^+^/CD44^+^ and EpCAM^+^/CD44^+^ cells	[47,49]
		Overexpression leads to enhanced formation of 3D organoids	
		Targets QKI which regulates LGR5, SOX2 and OCT-4	
		Anti-miR-221 inhibits CRC proliferation and promotes apoptosis	
miR-302a	Downregulated	Downregulated in cetuximab-resistant CRC ^1^	[58]
		Targets CD44	
miR-4711-5p	N/A	Inhibits proliferation of spheroid cells	[59]
		Targets CD44v9, LGR5, BMI1 and KLF5	

^1^ These miRNAs have demonstrated therapeutic potential.

**Table 2:** Summary of microRNAs (miRNAs) regulating pancreatic cancer stem cell (CSC) growth and/or stemness.

miRNA	Expression in CSCs	Relation to Stemness	Reference
miR-15a	Downregulated	Targets BMI-1	[75]
		5-FU-modified mimic promotes apoptosis, inhibits invasion and overcomes resistance in CSC ^1^	
miR-34	Downregulated	Targets c-Met	[74]
		Overexpression downregulates CD44 and CD133 and inhibits tumor formation in vivo	
		Enhances sensitivity to docetaxel and gemcitabine ^1^	
miR-135b	Upregulated	Upregulated in CD44^+^/CD24^+^/ESA^+^ cells	[76]
		Anti-miR-135b decreases expression of NANOG, ALDH1, SOX2 and OCT-4 in vitro and suppresses tumor growth in vivo ^1^	
miR-195	Downregulated	Targets DCLK1	[77]
miR-200a	Downregulated	Overexpression downregulates CD24, CD44, ESA, N-cadherin, ZEB1, and vimentin	[78,79]
		Upregulated after DCLK1 knockdown	
miR-205	Downregulated	Overexpression downregulates OCT-3/4, NANOG, CD44, and ALDH1	[80]
miR-1246	Upregulated	Upregulated in CD44^+^/CD24^+^ cells	[81]
		Overexpression increases tumor sphere-forming capability and resistance to gemcitabine	

^1^ These miRNAs have demonstrated therapeutic potential.

**Table 3:** Summary of microRNAs (miRNAs) regulating gastric cancer stem cell (CSC) growth and/or stemness.

miRNA	Expression in CSCs	Relation to Stemness	Reference
miR-26a	Downregulated	Targets HOXC9	[89]
		Overexpression reduces self-renewal capacity of spheroid cells and invasive capacity	
miR-106b	Upregulated	Upregulated in CD44^+^ cells	[87]
		Targets SMAD7	
miR-193-3p	Upregulated	Overexpression increases resistance to 5-FU and cisplatin^1^	[90]
miR-196a-5p	Upregulated	Upregulated in CD44^+^ cells	[88]
		Targets SMAD4	
miR-200a	Downregulated	Downregulated in ZEB1^+^ and Snail^+^, invasive cells	[91]
miR-200c	N/A	Delivery with micelle-based nanoparticles enhances sensitivity to radiotherapy ^1^	[92]
miR-216a-3p	Downregulated	Downregulated by BRD4	[93]
		Targets Wnt3a	
miR-524-5p	Downregulated	Enhances sensitivity to cisplatin ^1^	[94]
miR-876-3p	Downregulated	Enhances sensitivity to cisplatin ^1^	[95]
		Targets SMAD4	

^1^ These miRNAs have demonstrated therapeutic potential.

**Table 4:** Summary of microRNAs (miRNAs) regulating liver cancer stem cell (CSC) growth and/or stemness.

miRNA	Expression in CSCs	Relation to Stemness	Reference
miR-30e-3p	Downregulated	Downregulated in EpCAM^+^ cells	[107]
		Overexpression downregulates of AFP and EpCAM, and decreases spheroid colony formation	
miR-181	Upregulated	Upregulated in EpCAM^+^ and CD44^+^/CD24^+^/CD90^+^ cells	[108]
		Targets GATA6, CDX2, and NLK	
		Anti-miR-181 suppresses tumor growth in vivo ^1^	
miR-194	Downregulated	Downregulated in CD133^+^ and EpCAM^+^ cells	[109]
		Overexpression downregulates CD133, CD24, EpCAM, CD90, and spheroid cell number	
		Targets RAC1	
		Enhances sensitivity to sorafenib ^1^	
miR-221	N/A	Gold nanoparticles with anti-miR-221 enhance sensitivity to sorafenib ^1^	[110]
miR-589-5p	Upregulated	Targets SOCS2, SOCS5, PTPN1, and PTPN11	[111]
		Upregulates expression of CD133, NANOG, BMI-1, OCT-4 and SOX2	
		Promotes tumorigenic capacity in vivo	
		Anti-miR-589-5p enhances sensitivity to doxorubicin ^1^	
miR-613	Downregulated	Downregulated in CD24^+^/OV6^+^ cells	[112]
		Overexpression downregulates NANOG, OCT-4, and SOX2, and reduces formation of spheroid cells	
		Targets SOX9	
		Enhances sensitivity to sorafenib and cisplatin ^1^	
miR-1305	N/A	Targets UBE2T	[113]
		Overexpression inhibits spheroid formation in vitro and tumorigenic capacity in vivo ^1^	

^1^ These miRNAs have demonstrated therapeutic potential.

## Data Availability

Data sharing not applicable. No new data were created or analyzed in this study. Data sharing is not applicable to this article.
